# A Mediterranean lifestyle obesity prevention intervention in preschoolers at risk: MELI-POP Study—a randomized controlled trial

**DOI:** 10.1007/s00431-026-06844-3

**Published:** 2026-03-24

**Authors:** A. Larruy-García, P. De Miguel-Etayo, M. Gil-Campos, L. M. Esteban, R. Picáns-Leis, B. Pastor-Villaescusa, R. Vázquez-Cobela, K. Flores-Rojas, R. Leis, L. A. Moreno

**Affiliations:** 1 NUTRI-GENUD (B34_23R-Growth, Exercise, Nutrition and Development), Instituto de Investigación Sanitaria Aragón (IIS Aragón), Instituto Agroalimentario de Aragón (IA2), University of Zaragoza, Zaragoza, Spain; 2 Consorcio CIBER, Fisiopatología de La Obesidad y Nutrición (CIBEROBN), Instituto de Salud Carlos III (ISCIII), Madrid, Spain; 3 Metabolism and Investigation Unit, Maimonides Institute of Biomedicine Research of Córdoba (IMIBIC), Reina Sofia University Hospital, University of Córdoba, Córdoba, Spain; 4 Applied Mathematics Department, Escuela Universitaria Politécnica de La Almunia, Institute for Bio-Computation and Physics of Complex Systems, University of Zaragoza, Zaragoza, Spain; 5 Neonatology Service, RICORS-SAMID-CIBERER, University Clinical Hospital of Santiago de Compostela, Health Research Institute of Santiago de Compostela, Santiago de Compostela, Spain; 6 Primary Care Interventions to Prevent Maternal and Child Chronic Diseases of Perinatal and Developmental Origin (RICORS), Instituto de Salud Carlos III, RD21/0012/0008 Madrid, Spain; 7 Research Group of Pediatric Nutrition, Instituto de Investigación Sanitaria de Santiago (IDIS), Unidad de Gastroenterología, Hepatología y Nutrición Pediátrica del Hospital Clínico Universitario de Santiago, Santiago de Compostela, Spain; 8 Unit of Investigation in Nutrition, Growth and Human Development of Galicia, University of Santiago de Compostela, Santiago de Compostela, Spain

**Keywords:** Obesity, Mediterranean diet, Prevention, Intervention, Preschoolers

## Abstract

This study aimed to determine whether a Mediterranean lifestyle intervention, including diet and physical activity (PA), influences body composition and cardiovascular risk factors in preschoolers at risk of obesity due to family history. A parallel RCT was conducted in three Spanish cities, enrolling 206 children aged 3.0–6.9 years with normal weight or overweight and at least one parent with overweight or obesity. Participants were recruited from health centers, hospitals, and schools, and randomly assigned to either a Mediterranean lifestyle intervention or a control group. The intervention included provision of fish and olive oil, monthly nutrition education, and twice-weekly PA sessions, while the control group received general child health advice unrelated to diet or exercise. After 12 months, 170 children completed the study (17.5% dropout). Outcomes included BMI, fat mass index (FMI), and respective *z*-scores (primary), as well as waist-to-height ratio, blood pressure, lipid profile, glucose, and insulin (secondary). At baseline, no significant differences were observed between groups. Per-protocol analysis showed significant differences in BMI and FMI (kg/m^2^ and *z*-score) between the intervention and control groups among girls, but not among boys. Intention-to-treat analysis confirmed these findings in girls, showing significant changes in BMI (− 0.68 kg/m^2^) and BMI *z*-score (− 0.34) compared with the control group. In both per-protocol and intention-to-treat analyses, no significant between-group differences were observed in changes in cardiovascular risk factors.

*Conclusion*: A Mediterranean lifestyle intervention may benefit body composition changes in preschool girls at risk of obesity; larger studies are needed to confirm these sex-specific results.

*Trial registration*: ClinicalTrials.gov: NCT04597281. Date of register: 28–09-2020.https://www.clinicaltrials.gov/study/NCT04597281?id=NCT04597281&rank=1

**Table:** 

**What is Known:** • *Mediterranean diet-based interventions have shown benefits in reducing BMI and obesity in children and adolescents. While many studies focus primarily on BMI, fewer have reported detailed body composition measures or explored potential sex-specific effects.*	
**What is New:** • *This is the first randomized controlled trial evaluating the effectiveness and efficacy of a Mediterranean lifestyle prevention intervention—including diet and PA—in preschool children at risk of obesity. Including FMI and the FMI z-score enables more specific characterization of adiposity-related changes, which complement BMI-based outcomes.*

**Supplementary Information:**

The online version contains supplementary material available at 10.1007/s00431-026-06844-3.

## Introduction

Childhood obesity is a global health concern due to its high prevalence [1, 2], early-onset complications [3, 4], limited treatment efficacy [5], and long-term economic burden [6]. Most prevention strategies, especially from preschool age, focus on lifestyle changes: promoting physical activity (PA), reducing sedentary behaviors, improving sleep, and enhancing diet quality [7, 8] potentially avoiding early adiposity rebound, and later obesity development [9, 10].

Mediterranean diet (MD) is considered one of the healthiest dietary patterns, especially for cardiovascular prevention [11]. This varied diet promotes the consumption of fresh, local, and seasonal foods [12]. It is primarily defined by a high intake of vegetables, fruits, and legumes, which provide antioxidants and fiber, olive oil, nuts, and fish that serve as the main sources of mono- and polyunsaturated fatty acids [13]. In children, there is scarce information about the association between the MD and body composition or cardiovascular risk factors. The majority of the available observational studies show an inverse association between MD and obesity [14, 15]. A systematic review and meta-analysis in children aged 3 to 18 years in Mediterranean countries showed that dietary interventions significantly reduced the body mass index (BMI), despite small samples, short durations, and targeting older children and adolescents [16]. Concerning PA, another systematic review found strong evidence that high levels of PA in children younger than 6 years are associated with better adiposity indicators, although evidence on cardiometabolic health was limited [17]. A recent update to the MD pyramid for children and adolescents recommends promoting this diet combined with PA [18].

Studies on obesity prevention in children and young people in school settings have shown heterogeneous results [19]. The European IDEFICS study found a significant effect in children with overweight or obesity at baseline, but not on obesity prevention among children with normal weight [20], possibly due to shared genetic or environmental factors; the intervention in this study was carried out on all children in the classrooms, regardless of whether they had overweight or obesity, supporting the idea that obesity prevention interventions should focus not on the general population, but on at-risk populations [21]. However, there is a very limited number of studies focusing on children or adolescents at risk of developing obesity [22]. Therefore, our study aims to evaluate the efficacy and effectiveness of a Mediterranean lifestyle intervention to prevent adiposity increase in preschool children at risk of obesity after 1 year of intervention.

## Materials and methods

### Study design

The MELI-POP (MEditerranean Lifestyle In Pediatric Obesity Prevention) Study is a multi-center, parallel, randomized, and controlled clinical trial performed in three Spanish cities (Córdoba, Santiago de Compostela, and Zaragoza), including children aged 3–6 years at baseline who are at risk of developing obesity because one or both parents were overweight or obese at the time of recruitment. Recruitment was performed in hospitals, health care centers, or by researchers at nearby schools in the same health areas as the Primary Health Care centers. This study is planned to have a follow-up period of 10 years.

A 2-week run-in period with two visits was conducted to engage families and minimize dropouts. Families meeting inclusion criteria and completing this phase were randomized into the corresponding study groups.

Inclusion criteria: children aged 3.0 to 6.9 years, having normal weight or overweight according to sex and age-specific cut-offs of Cole et al. standards [23] and at least one parent with a BMI ≥ 25 kg/m^2^, excluding chronic conditions causing obesity or pharmacological treatments influencing body weight. Exclusion criteria: children with chronic diseases, following a therapeutic diet or any other regimen incompatible with the study’s dietary intervention.

This study is registered in ClinicalTrials.gov (ID: NCT04597281). The study protocol has already been described in detail [24]. It has been approved by the Ethics Committee of each recruitment center (references: 3669—Institutional Hospital Ethics Committee (Córdoba), 2017/501—Santiago-Lugo Committee of Ethics in Clinical Research (Santiago de Compostela), PI17/0338—Aragon Committee of Ethics in Clinical Research (Zaragoza)) and conducted following the standards of the Declaration of Helsinki and further updates. Both parents or legal tutors were required to sign the informed consent.

### Randomization

Between 1 and 3 weeks after the run-in period was concluded, participants underwent random assignment to either the control or intervention group. Randomization was conducted per intervention center via a centralized computer system, Sealed Envelope (www.sealedenvelope.com), by block randomization of a fixed size of 8. Once assigned, group allocation was not altered. To facilitate the study procedure for the families, siblings were allocated as family units in the same group.

### Intervention

The main goal of the intervention was to promote a Mediterranean lifestyle (MD and regular PA). The intervention was conducted by a multidisciplinary team including dieticians-nutritionists, nurses, pediatricians, and PA experts. The intervention focuses on both parents and children, so that they can replicate everything they learn in the sessions at home. The sessions include detailed information on the Mediterranean lifestyle (diet and PA). The intervention group attended monthly group sessions on different aspects of the Mediterranean dietary pattern. Every fourth month, an individual monitoring session was conducted during which families received extra-virgin olive oil and fish to be consumed daily and twice a week, respectively, for the entire family; these foods were offered for free. During these sessions, adherence to the MD and PA was assessed and new goals were set to further improve lifestyle habits. Twice a week, children were offered a guided PA session at no cost to the family. The sessions were of 1 h duration each and focused on achieving a moderate-to-vigorous intensity; heart rate monitors ensured that training intensity was appropriate. Activities (including games and exercises) were adapted to the age and psychomotor level of the participants.

The control group had a single monitoring visit in month 6, receiving general guidelines on childcare, road safety, or accident prevention, topics that were not directly related to diet or PA.

### Study sample

The sample size was estimated based on the main objective (changes in BMI *z*-score) and the expected effect size. A previous study in preschoolers with normal weight or overweight over 6–8 months reported a BMI *z*-score reduction of 0.14 in the control group and 0.27 in the intervention group, with a between-group difference of 0.13 [25]. According to this information, the expected difference in BMI *z*-score changes between groups in our study is 0.20. Considering a power of 0.95, an *α*-error of 0.05, and an expected dropout loss of 15%, the required sample size was calculated to be 300 participants.

**Fig. 1:**
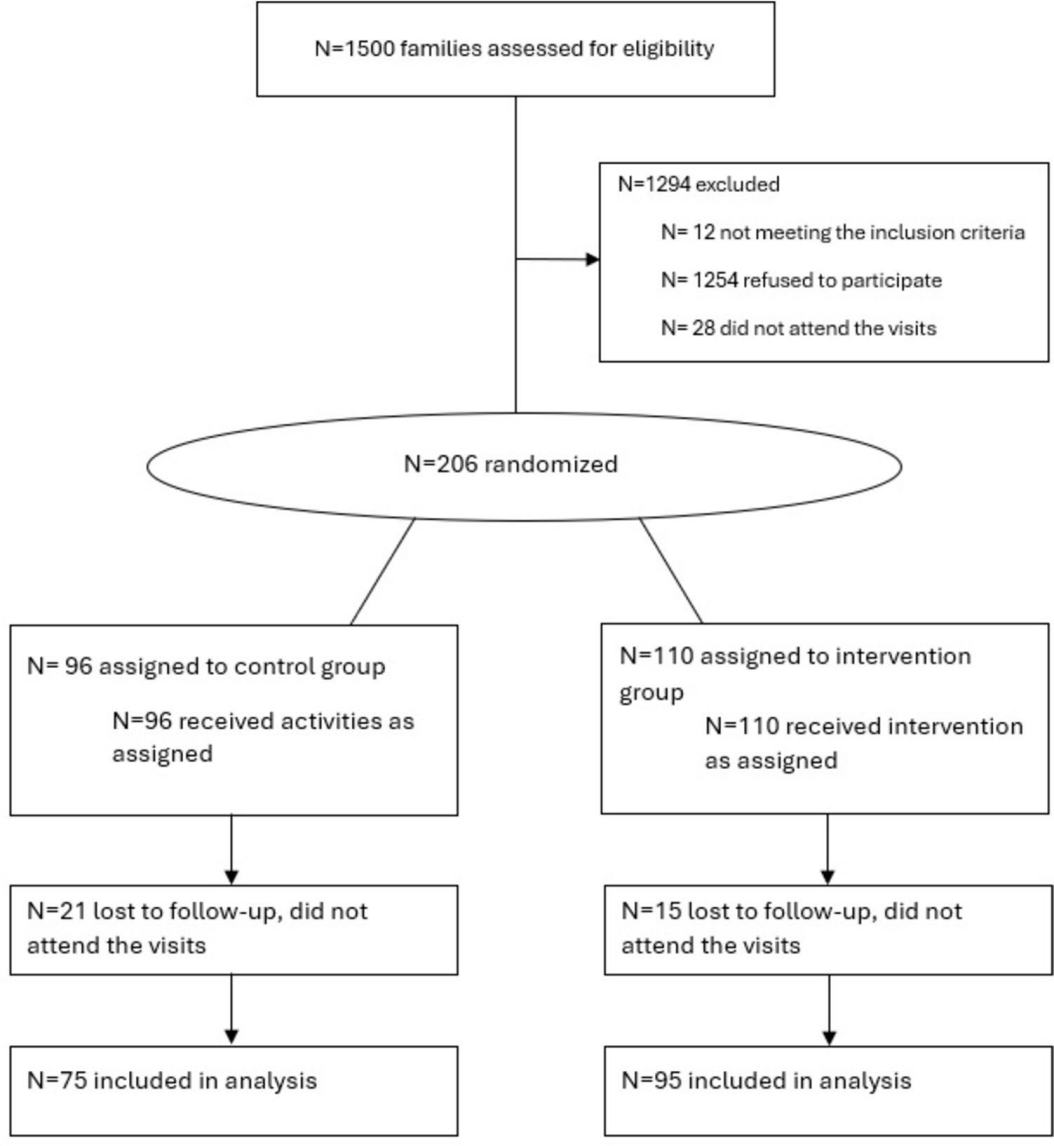
Flowchart of the MELIPOP Study

Of 1.500 families contacted, 1244 declined to participate or did not attend the first visit. The remaining 256 met the inclusion criteria. A run-in period consisted of two visits separated by 2 weeks, in which families were asked to fill in initial questionnaires as described elsewere [24], to learn about the child’s habits and history. If the families complied with the attendance and completion of these questionnaires, children were included in the trial and randomization was performed. After the run-in period, 206 (50.9% girls) accepted to participate and were then randomized. A total of 170 (75 control, 95 intervention) of them completed the 12-months intervention including anthropometric measurements and blood samples (dropout rate of 17.5%) (Fig. 1). With this information, the power calculation was updated, indicating that the study has a power greater than 80% to detect the same effect size.

### Main outcome variables

Body weight and body composition measures were obtained by trained researchers using a TANITA device (MC780SMA, Tanita Europe, B.V.). Height (cm) was measured using a portable stadiometer (SECA 213, Scale 20–205 cm; SECA). Waist circumference (WC, cm) was determined using a measuring tape (Cescorf, 1 mm accuracy) following the ISAK protocol [26]. The calculated variables included BMI as weight (kg) divided by height squared (m^2^), fat mass index (FMI) [27], and fat-free mass index (FFMI) [27] as fat mass and fat-free mass (kg), respectively, divided by height squared (m^2^), and waist-to-height ratio (WHtR) as WC (cm) divided by height (cm). BMI categories and sex- and age-normalized scores of BMI were calculated according to Cole et al. [23], and FMI and FFMI based on Nagy et al. [28].

### Secondary outcomes

Blood pressure was measured twice on the non-dominant arm using an OMRON oscillometer (Omron M3 Intellisense HEM-75051-EV; IOMRON Healthcare Europe) with a child-specific cuff to ensure accuracy.

Biochemical analyses were performed by standardized laboratory methods using Architect c16000 and i2000SR autoanalyzers (Abbott Diagnostics®, Abbott Laboratories, Madrid, Spain). They included glucose and lipid profile (plasma total cholesterol, low-density lipoprotein cholesterol (LDL-C), high-density lipoprotein cholesterol (HDL-C), and triglycerides). Plasma insulin was analyzed by radioimmunoassay (CV = 2.6%) using an automatic microparticle analyzer (AxSYM; Abbott Laboratories, Abbott Park, IL, USA). C-reactive protein (CRP) was analyzed by turbidimetric immunoassay with latex particles. External and internal quality controls were performed according to hospital protocols. Insulin resistance was assessed by the Homeostatic Model Assessment for Insulin Resistance index (HOMA-IR) calculated as insulin (mU/L) × glucose (mmol/L)/22.5 [29].

The covariates used to adjust the analyses were as follows: the children’s age at baseline, the mother’s level of education (reported by the parents), MD adherence (validated MED4CHILD questionnaire [30]), total energy intake (validated COME-Kids Food and Beverage Frequency Questionnaire [31]), and total minutes of PA per week (obtained by a PA questionnaire, developed from the questionnaires used at European level; an active lifestyle is assessed using the Outdoor Playtime Checklist and the Outdoor Playtime Recall Questions [32]).

### Statistical analysis

In this study, we conducted the 12-month analysis of the clinical trial. Study variables were presented as *n* and % for categorical variables, mean ± SD and median (interquartile range) for quantitative variables depending on whether or not they were normally distributed, at the beginning and 12-month intervention.

The crude change for each variable was estimated as the difference between the median variation for the intervention group and the median variation for the control group. Although the original protocol planned to conduct analyses on the total sample, observed interactions with sex prompted additional post hoc analyses stratified by sex; thus, the analysis was ultimately conducted separately for boys and girls [33, 34]. To assess the efficacy of the intervention, we performed per-protocol analysis in 170 children who completed the intervention. To assess the effectiveness of the intervention, we performed intention-to-treat analysis in the total sample of 206 children. To perform intention-to-treat analysis (effectiveness), missing data were imputed. This imputation was conducted through joint modeling with a random forest approach. Maternal educational level and adherence to the MD were considered as covariates, because they could influence the study’s outcome and complete information was available at baseline.

To evaluate the effect size of the intervention based on a per-protocol analysis, mixed linear models were developed to predict changes in the study variables based on initial characteristics, including age, energy intake, total PA, and the categorical variable distinguishing intervention from control (ref.). In the mixed linear model analyses, the center was included as a random effect to account for between-center variability. The effect of the intervention was quantified using its estimated coefficient within the model, alongside the corresponding 95% confidence intervals (CI) and *p*-value. These results are presented graphically using a forest plot. The same analysis was performed with the imputed data (intention-to-treat).

The level of statistical significance was fixed as *p* < 0.05. All calculations were performed using the R statistical programming language (version 4.4.0; The R Foundation for Statistical Computing, Vienna, Austria), with the additional libraries sciplot and missForest.

## Results

At baseline, no statistically significant differences were observed between intervention and control groups (Table Supplementary 1). Descriptive analysis of baseline data of the complete sample and data of those completing the 12-month intervention in the intervention and control groups is shown in Table 1. Table 1 shows that 76.5% of participants in the intervention group and 74.7% in the control group presented adherence to the MD at baseline, whereas after 12 months, these values were 94.8% in the intervention group and 66.7% in the control group. There is also a small increase in BMI among participants in the intervention group. Per-protocol analysis results are shown in Supplementary Tables 2 (for girls) and 3 (for boys), showing significant differences in BMI and FMI (kg/m^2^ and *z*-score) changes in the intervention compared with the control group for girls, but not for boys.

**Table 1:** Descriptive analysis of baseline data of the complete sample and data of those completing the 12-month intervention in the intervention and control groups

	Baseline		12-month intervention	
	Intervention (*n* = 110)	Control (*n* = 96)	Intervention (*n* = 95)	Control (*n* = 75)
General characteristics				
Sex (girls)	56 (50.9%)	47 (49.0%)	47 (49.5%)	37 (49.4%)
Age	4.96 ± 1.16	5.06 ± 1.00	6.23 ± 1.20	6.30 ± 1.08
BMI of parent with OW/OB (kg/m^2^)	32.27 ± 5.89	32.40 ± 4.95	-	-
Mother education level*				
Low	13 (12.0%)	4 (4.3%)	-	-
Medium	46 (42.6%)	40 (43.0%)	-	-
High	47 (43.5%)	45 (48.4%)	-	-
Mediterranean diet adherence score	11.53 ± 2.37	11.34 ± 2.75	13.01 ± 2.31	10.84 ± 2.82
Mediterranean diet adherence**				
Adherence (≥ 10 points)	78 (76.5%)	65 (74.7%)	90 (94.8%)	50 (66.7%)
Non-adherence (< 10 points)	24 (23.5%)	22 (25.3%)	5 (5.2%)	25 (33.3%)
Total energy intake (kcal/day)	1581.54 ± 353.64	1663.94 ± 439.07	1553.72 ± 434.97	1589 ± 422.40
Physical activity (min/week)	183.83 ± 112.41	219.85 ± 163.05	268.22 ± 154.92	254.44 ± 137.73
Anthropometric and body composition				
BMI (kg/m^2^)	16.22 (15.47, 17.12)	16.01 (15.42, 16.97)	16.41 (15.43, 17.55)	16.68 (15.79, 17.70)
BMI *z*-score	0.35 (− 0.19, 0.99)	0.30 (− 0.19, 0.86)	0.57 (− 0.06, 1.15)	0.64 (0.14, 1.34)
BMI categories				
Under/normal weight	90 (81.8%)	79 (82.3%)	69 (72.6%)	54 (72.0%)
Overweight	20 (18.2%)	17 (17.7%)	22 (23.2%)	14 (18.7%)
Obesity	-	-	4 (4.2%)	7 (9.3%)
FMI (kg/m^2^)	3.56 (3.00, 4.43)	3.52 (3.20, 4.01)	3.61 (3.07, 4.45)	3.74 (3.34, 4.77)
FMI *z*-score	1.61 (1.17, 2.65)	1.63 (1.16, 2.26)	1.65 (1.06, 2.52)	1.88 (1.39, 3.06)
FFMI (kg/m^2^)	12.13 (11.61, 12.80)	11.80 (11.40, 12.56)	12.08(11.70, 12.88)	12.32 (11.64, 13.26)
FFMI *z*-score	− 1.09 (− 1.69, − 0.62)	− 1.20 (− 1.79, − 0.68)	− 0.12 (− 0.62, 0.69)	− 0.08 (− 0.54, 0.72)
WC (cm)	52.5 (50.4, 55.0)	52.20 (49.50, 54.70)	55.00 (52.74, 58.35)	55.20 (52.50, 58.35)
WHtR	0.48 (0.45, 0.51)	0.48 (0.45, 0.51)	1.25 (0.62, 2.09)	1.11 (0.69, 2.06)
WHtR *z*-score	0.16 (− 0.26, 0.75)	0.22 (− 0.56, 0.79)	1.15 (0.40, 1.58)	1.00 (0.21, 1.92)
Cardiovascular risk factors				
SBP (mmHg)	102.00 (97.00, 108.50)	102.00 (95.00, 109.00)	102.50 (95.75, 109.00)	104.25 (96.62, 111.00)
DBP (mmHg)	62.00 (57.00, 68.00)	61.00 (55.50, 68.50)	61.00 (57.00, 68.00)	62.75 (58.25, 68.00)
Total cholesterol (mg/dL)	161.0 (150.0, 182.0)	163.0 (144.2, 183.5)	166.0 (144.5, 184.0)	167.0 (149.8, 190.2)
HDL-C (mg/dL)	54.0 (46.0, 63.0)	55.0 (47.5, 62.0)	58.0 (49.0, 66.0)	58.50 (50.00, 67.25)
LDL-C (mg/dL)	96.00 (85.00, 102.60)	97.00 (77.00, 112.45)	95.0 (78.0, 111.7)	97.0 (80.5, 111.0)
Triglycerides (mg/dL)	54.00 (46.00, 70.00)	54.0 (44.0, 65.0)	55.0 (41.5, 68.5)	56.0 (46.0, 75.0)
Glucose (mg/dL)	81.0 (77.00, 87.00)	83.00 (75.75, 87.00)	84.0 (79.0, 88.0)	84.0 (79.5, 88.0)
Insulin (uU/mL)	3.35 (2.00, 5.16)	3.80 (2.00, 5.80)	3.09 (2.00, 5.37)	3.76 (2.00, 7.05)
HOMA-IR	0.62 (0.44, 1.02)	0.67 (0.41, 1.22)	0.64 (0.42, 1.21)	0.72 (0.42, 1.51)
CRP (mg/dL)	0.06 (0.02, 0.19)	0.08 (0.02, 0.36)	0.08 (0.03, 0.50)	0.05 (0.03, 0.25)

Values are presented as *n* and % for categoric variables, mean ± SD and median (interquartile range) for quantitative variables depending on their distribution as normal or not normal. BMI categories according to Cole et al. criteria [19]

*BMI* body mass index, *CRP* C-reactive protein, *DBP* diastolic blood pressure, *FMI* fat mass index, *FFMI* fat free mass index, *HDL-c* high-density lipoprotein cholesterol, *HOMA-IR* homeostatic model assessment of insulin resistance, *LDL-c* low-density lipoprotein cholesterol, *OB* obesity, *OW* overweight, *SBP* systolic blood pressure, *WC* waist circumference, *WHtR* waist-to-height ratio

^*^Classification of the education level according to ISCED

^**^Mediterranean diet adherence evaluated through validated MED4CHILD questionnaire [25]

Normalized root mean square (NRMSE) values in the imputation process are shown in Supplementary Table 4. Lower values indicate better imputation accuracy.

**Fig. 2:**
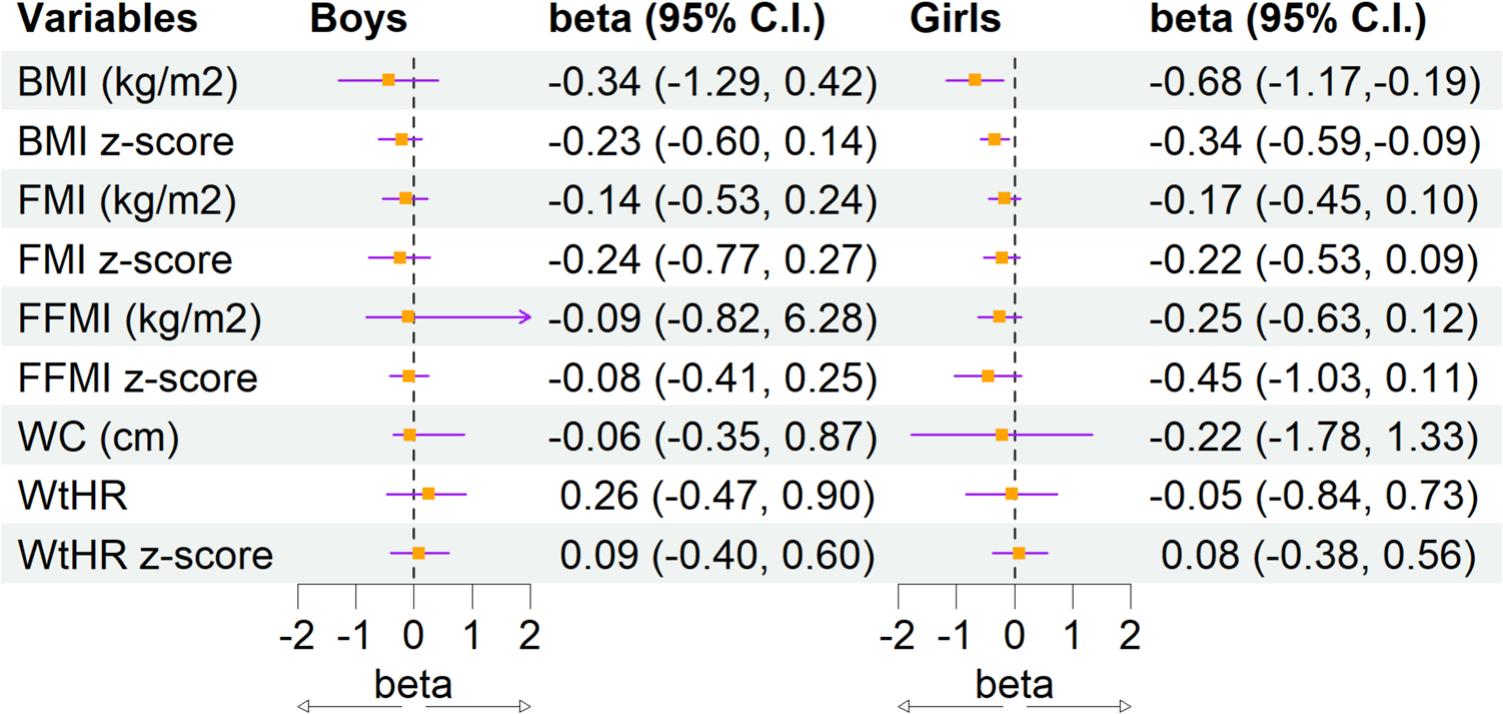
Forest plot displaying effectiveness (intention-to-treat) analysis of Mediterranean lifestyle intervention on body composition parameters. The dashed vertical line at zero represents no effect, and markers to the left or right indicate directionality of associations. For boys, the beta estimates were close to zero, indicating negligible differences between groups. For girls, the beta values for BMI and BMI *z*-score showed a significant (*p* < 0.003) effect of the intervention (− 0.68 kg/m^2^ (− 1.17, − 0.19) kg/m^2^ and − 0.34 (− 0.59, − 0.09), respectively), consistent with the per-protocol analysis, reinforcing their robustness. Other body composition variables in girls show beta estimates near to zero, with no statistically significant differences. BMI, body mass index; FMI, fat mass index; FFMI, fat free mass index; WC, waist circumference; WtHR, waist-to-height ratio

Effectiveness (intention-to-treat analysis) of Mediterranean lifestyle intervention on body composition in boys and girls is shown in Fig. 2.

For the remaining variables, data of the intention-to-treat analysis are presented in Tables 2 and 3 showing that the intervention had no effect on the measured cardiovascular parameters in either sex, as evidenced by small effect sizes and non-significant *p*-values. For systolic blood pressure (SBP), diastolic blood pressure (DBP), glucose, lipid profile components (total cholesterol, HDL-C, LDL-C, and triglycerides), insulin levels, HOMA-IR, and CRP, the crude changes from baseline to the end of the 12-month intervention were comparable between the intervention and control groups. None reached statistical significance (*p* > 0.05), indicating no changes in initially normal-range parameters.

**Table 2:** Effectiveness (intention-to-treat) analysis of Mediterranean lifestyle intervention on cardiovascular risk factors in girls

	Baseline		12-month intervention		∆ Intervention	∆ Control	Crude change	Effect size (intervention vs control)	
	Intervention (*n* = 56)	Control (*n* = 47)	Intervention (*n* = 56)	Control (*n* = 47)				Estimate	*p*-value
SBP (mmHg)	101.50 (96.98, 105.81)	100.40 (93.50, 105.5)	101.80 (97.00, 106.62)	103.00 (97.00, 108.50)	0.30	2.60	0.825	− 0.25 (− 3.88, 3.37)	0.902
DBP (mmHg)	63.20 (56.75, 68.00)	61.34 (54.50, 66.50)	64.00 (59.00, 68.12)	64.14 (60.00, 68.00)	0.80	2.80	− 1.618	0.76 (− 2.57, 4.11)	0.643
Total cholesterol (mg/dL)	163.9 (150.9, 176.6)	164.00 (152.0, 179.9)	164.4 (148.5, 180.8)	168.2 (154.6, 179.0)	0.5	4.2	1.187	− 4.90 (− 14.69, 4.88)	0.304
HDL-C (mg/dL)	54.33 (49.00, 59.00)	54.72 (48.50, 60.27)	56.23 (50.50, 60.25)	56.33 (50.59, 61.50)	1.90	1.61	− 1.000	0.80 (− 2.72, 4.32)	0.662
LDL-C (mg/dL)	98.23 (85.50, 103.58)	96.45 (79.50, 108.00)	94.88 (78.65, 107.80)	97.84 (84.90, 107.13)	− 3.35	1.39	1.953	− 6.13 (− 14.80, 2.53)	0.168
Triglycerides (mg/dL)	62.37 (49.75, 60.67)	63.42 (48.50, 67.50)	66.55 (51.75, 72.20)	70.31 (55.00, 74.36)	4.18	6.89	2.949	− 1.32 (− 11.83, 9.18)	0.779
Glucose (mg/dL)	79.81 (75.88, 85.00)	78.46 (74.00, 84.50)	83.40 (80.58, 86.96)	81.59 (78.63, 86.00)	3.59	3.13	0.772	1.08 (− 2.00, 4.18)	0.495
Insulin (uU/mL)	5.06 (3.25, 5.71)	5.88 (3.68, 7.20)	5.29 (2.72, 6.00)	6.44 (3.09, 6.31)	0.23	0.56	− 0.090	− 1.27 (− 3.78, 1.23	0.348
HOMA-IR	1.01 (0.59, 1.25)	1.15 (0.65, 1.34)	1.15 (0.54, 1.34)	1.41 (0.66, 1.37)	0.14	0.26	− 0.034	− 0.31 (− 0.93, 0.31)	0.367
CRP (mg/dL)	0.83 (0.03, 0.56)	0.37 (0.03, 0.41)	0.68 (0.07, 0.53)	0.45 (0.10, 0.50)	− 0.15	0.08	0.007	0.42 (− 0.21, 1.06)	0.166

Values are presented as median (interquartile range). The level of statistical significance was fixed as *p* < 0.05

Analysis was adjusted by mother’s level of education, adherence to the Mediterranean diet in baseline, total energy intake, and minutes of physical activity per week

*∆* changes, *CRP* C-reactive protein, *DBP* diastolic blood pressure, *HDL-c* high-density lipoprotein cholesterol, *HOMA-IR* homeostatic model assessment of insulin resistance, *LDL-c* low-density lipoprotein cholesterol, *SBP* systolic blood pressure

**Table 3:** Effectiveness (intention-to-treat) analysis of Mediterranean lifestyle intervention on cardiovascular risk factors in boys

	Baseline		12-month intervention		∆ Intervention	∆ Control	Crude change	Effect size (intervention vs control)	
	Intervention (*n* = 54)	Control (*n* = 49)	Intervention (*n* = 54)	Control (*n* = 49)				Estimate	*p*-value
SBP (mmHg)	102.9 (98.24, 109.38)	103.7 (98.00, 110.0)	103.3 (97.25, 109.50)	103.8 (100.00, 109.00)	0.4	0.1	− 2.248	− 0.38 (− 4.81, 4.05)	0.864
DBP (mmHg)	63.76 (59.25, 69.50)	66.09 (58.5, 73.00)	60.98 (55.50, 63.04)	62.30 (59.00, 65.00)	− 2.78	− 3.79	− 1.000	− 1.86 (− 5.15, 1.43)	0.263
Total cholesterol (mg/dL)	163.0 (151.2, 179.5)	162.3 (147.9, 176.0)	166.8 (151.0, 181.2)	170.8 (157.0, 182.5)	3.8	8.5	− 0.261	− 4.16 (− 13.88, 5.55)	0.395
HDL-C (mg/dL)	56.08 (48.25, 63.00)	56.17 (50.0, 61.0)	62.45 (52.25, 66.00)	62.40 (57.00, 67.00)	6.37	6.23	− 0.087	0.34 (− 4.26, 4.94)	0.843
LDL-C (mg/dL)	95.44 (86.25, 102.26)	95.88 (82.00, 109.20)	95.79 (85.05, 107.86)	97.35 (88.59, 105.65)	0.35	1.47	− 1.200	− 1.59 (− 9.12, 5.92)	0.656
Triglycerides (mg/dL)	55.86 (46.72, 58.43)	53.2 (44.0, 60.0)	53.52 (40.25, 61.93)	56.74 (46.00, 66.00)	− 2.34	3.54	3.202	− 2.15 (− 8.67, 4.36)	0.451
Glucose (mg/dL)	82.72 (78.00, 88.06)	83.67 (80.00, 88.06)	83.53 (78.25, 88.75)	85.23 (82.00, 90.00)	0.81	1.56	0.274	− 1.11 (− 3.82, 1.58)	0.374
Insulin (uU/mL)	3.47 (2.24, 4.02)	3.52 (2.41, 3.74)	4.27 (2.92, 5.48)	4.71 (2.80, 6.00)	0.80	1.19	0.487	− 0.13 (− 1.00, 0.74)	0.703
HOMA-IR	0.72 (0.47, 0.82)	0.75 (0.50, 0.78)	0.91 (0.62, 1.19)	1.02 (0.65, 1.21)	0.19	0.27	0.134	− 0.02 (− 0.22, 0.16)	0.804
CRP (mg/dL)	0.86 (0.03, 0.64)	0.84 (0.03, 0.69)	0.60 (0.04, 0.59)	0.40 (0.04, 0.59)	− 0.26	− 0.44	0.017	0.30 (− 0.10, 0.72)	0.141

Values are presented as median (interquartile range). The level of statistical significance was fixed as *p* < 0.05

Analysis was adjusted by mother’s level of education, adherence to the Mediterranean diet at baseline, total energy intake, and minutes of physical activity per week

*∆* change, *CRP* C-reactive protein, *DBP* diastolic blood pressure, *HDL-c* high-density lipoprotein cholesterol, *HOMA-IR* homeostatic model assessment of insulin resistance, *LDL-c* low-density lipoprotein cholesterol, *SBP* systolic blood pressure

There were no study-related adverse events.

## Discussion

The MELI-POP Study assesses the efficacy and effectiveness of a Mediterranean lifestyle intervention to maintain adequate BMI and FMI in preschoolers. Effectiveness was assessed by imputing outcome variables based on maternal educational level and baseline MD adherence as covariates. Because randomization was performed by center, in order to ensure balanced groups within each city, the number of children in each study arm differed slightly. In girls, intervention was effective with significant BMI and BMI *z*-score changes (− 0.68 kg/m^2^ and − 0.34, respectively), while no significant differences were observed in boys. Per-protocol efficacy analysis showed significant differences in BMI (kg/m^2^ and *z*-score), and FMI (kg/m^2^ and *z*-score), specifically in girls. To our knowledge, this is the first study assessing the effectiveness of a Mediterranean lifestyle intervention to prevent adiposity increase in preschoolers. In girls, both the per-protocol and intention-to-treat analyses showed the expected changes in BMI and BMI *z*-score. Despite significant results for FMI and FMI *z*-score being only observed in the per-protocol analysis, these results suggest that the effect on BMI may be linked to changes in fat mass. This interpretation is consistent with evidence from large longitudinal studies, such as the IDEFICS/I.Family cohort, which showed that changes in BMI *z*-score are more strongly associated with changes in FMI than with fat-free mass index, particularly among children with overweight, obesity, or excessive weight gain trajectories [35].

The main outcome of the study was BMI *z*-score; however, we also assessed other body composition indices and some cardiovascular variables. BMI has well-recognized limitations, as it does not distinguish between fat mass and fat-free mass [36]. Body composition was assessed not only by measuring weight and height but also using bioelectrical impedance, allowing the estimation of fat mass and fat-free mass. In the per-protocol analysis, a significant intervention effect was observed for FMI, supporting the interpretation that the observed effect on BMI is likely driven by changes in fat mass [37]. The impact on overweight and obesity percentages has not yet been assessed due to short assessment.

A systematic review identified 15 Diet-Based interventions in Mediterranean countries [16]; however, two of them [38, 39] did not mention the MD as an intervention component, but general nutrition interventions. Only four included preschoolers [39–42], eight included participants of all weight categories, and seven included only participants with excess weight and were not comparable with our study, in which preschool children had normal weight or overweight. The vast majority include indices such as BMI, WC, or WHtR, but none has used indices like FMI or FFMI, as in our study.

Studies assessing MD adherence and its effects on body composition in preschoolers have been conducted over a wide range of intervention durations, typically ranging from several months up to 2 years. Most report outcomes after 6–12 months, but such brief follow-ups limit evaluation of long-term effects and sustained adherence [16]. Another RCT evaluated the efficacy of a MD intervention among preschoolers and found that the intervention led to a reduction in BMI in the experimental group after 1 year and at the end of the 36-month follow-up period [42]. Despite this study performed only per-protocol analysis (efficacy), their findings align with our results, indicating that Mediterranean diet-based interventions can effectively improve body composition in preschool children. However, while their study focused exclusively on BMI, we also examined BMI *z*-scores and other body composition indices, providing a more comprehensive assessment of body composition changes. Whereas they did not analyze outcomes by sex, our findings highlight notable sex differences, underscoring the importance of considering sex-specific effects in interventions targeting body composition in early childhood. A school-based intervention in preschoolers showed significant differences between intervention and control groups in glucose, total cholesterol, HDL-c, LDL-c, and triglycerides [40]. Our study included children with normal and overweight statuses, with cardiovascular risk factors within normal ranges at baseline; therefore, we did not observe significant differences in their changes, and this can be considered a positive finding. Because most participants started from healthy baseline values, the scope for measurable improvement was limited, reflecting a ceiling effect commonly observed in prevention trials. This suggests that the intervention primarily contributed to maintaining values within normal ranges, rather than producing marked changes. Evidence suggests that Mediterranean diet-based interventions can contribute to significant reductions in BMI and obesity in children and adolescents [16].

There is only one study assessing the efficacy of a Mediterranean lifestyle intervention (diet, PA), but it was performed in adolescents; there is no one in preschool or school-age children. The study observed that overall adherence to the MD was associated with more favorable body composition, including body fat levels [43]. These findings echo our results for BMI and FMI but only in girls, suggesting the need for sex-focused strategies in future interventions.

### Limitations and strengths

The sample size in our study is not very large due to the difficulty of recruiting participants who meet the characteristics and are committed. However, the power of the sample was sufficient to capture the expected effect after 12 months of intervention, considering most previous studies on MD interventions were conducted in smaller samples [16]. The provision of extra-virgin olive oil and fish could represent a limitation in terms of scalability of the strategy due to limited availability and elevated cost. The dropout rate can be considered another limitation. After 12 months of intervention, 17.5% of the children dropped out of the study, 13.6% from the intervention group, and 21.8% from the control group. The lower dropout in the intervention group could be attributed to closer monitoring of intervention families, the provision of extra-virgin olive oil and fish during clinical visits, and the encouragement of regular PA. Nonetheless, the dropout rate was similar to the studies only including the dietary component (ranging 0 to 42% [44]). Although the MED4CHILD questionnaire is validated for assessing children’s adherence to the Mediterranean diet, the limitations associated with parent-reported dietary questionnaires, including recall bias and social desirability bias, should be acknowledged.

The duration of our study can be considered long term; most previous studies had shorter follow-ups, ranging from 8 weeks to 36 months [16]. In contrast, the present study shows the first-year results of the intervention. Extended follow-ups are crucial to capturing delayed effects often missed in shorter studies. Findings will contribute valuable insights into the long-term effectiveness of the MD and its impact on development and weight-related risk reduction in children.

## Conclusions

The first-year findings of the MELI-POP Study on the effectiveness of a Mediterranean lifestyle intervention, incorporating diet and PA in preschool children at risk of obesity, revealed meaningful outcomes. Significant differences between the intervention and control groups were observed for BMI and FMI (only in per-protocol analysis) changes among girls, while no significant changes were noted in boys for any of the measured parameters, including body composition, blood pressure, or cardiovascular markers. These results also suggest potential sex-specific responsiveness to lifestyle interventions at this early age.

Extended assessments and tailored approaches are required to better understand and enhance the impact of Mediterranean lifestyle interventions in preschool-aged children.

## Supplementary Information

Below is the link to the electronic supplementary material.

Supplementary file 1 (DOCX 50.1 KB)

## Data Availability

The datasets generated and analyzed during the current study are not publicly available due to data regulations and for ethical reasons, considering that this information might compromise research participants’ acceptance because our participants only gave their consent for the use of their data by the original team of investigators. However, collaborations for data analyses can be requested by sending a letter to the corresponding author. The request will then be passed to all the members of the MELI-POP Steering Committee for deliberation.

## Abbreviations

BMI: Body mass index
CRP: C-reactive protein
DBP: Diastolic blood pressure
FMI: Fat mass index
FFMI: Fat-free mass index
HDL-C: High-density lipoprotein cholesterol
HOMA-IR: Homeostatic Model Assessment for Insulin Resistance Index
LDL-C: Low-density lipoprotein cholesterol
MELI-POP: MEditerranean Lifestyle In Pediatric Obesity Prevention
PA: Physical activity
SBP: Systolic blood pressure
WC: Waist circumference
WHtR: Waist-to-height ratio
