# Supplementary material for: A Mediterranean lifestyle obesity prevention intervention in preschoolers at risk: MELI-POP Study—a randomized controlled trial
Source: Eur J Pediatr. 2026 Mar 24;185(4):207. doi: 10.1007/s00431-026-06844-3 (PMC13013194; doi:10.1007/s00431-026-06844-3)
Supplement: Supplementary file 1 — (DOCX 50.1 KB) [file 431_2026_6844_MOESM1_ESM.docx]

Table Supplementary 1. Descriptive analysis of baseline.

|  | Intervention (n=110) | Control (n=96) | P value |
| --- | --- | --- | --- |
| BMI (kg/m^2^) | 16.22 (15.47,17.12)  n=110 | 16.01 (15.42,16.97)  n=96 | 0.404 |
| BMI z-score | 0.35 (-0.19,0.99)  n=110 | 0.30 (-0.19,0.86)  n=96 | 0.401 |
| FMI (kg/m^2^) | 3.56 (3.00,4.43)  n=73 | 3.52 (3.20,4.01)  n=68 | 0.951 |
| FMI z-score | 1.61 (1.17,2.65)  n=73 | 1.63 (1.16,2.26)  n=68 | 0.891 |
| FFMI (kg/m^2^) | 12.13 (11.61,12.80)  n=73 | 11.80 (11.40,12.56)  n=68 | 0.237 |
| FFMI z-score | -1.09 (-1.69,-0.62)  n=95 | -1.20 (-1.79,-0.68)  n=83 | 0.314 |
| WC (cm) | 52.5 (50.4,55.0)  n=101 | 52.20 (49.50,54.70)  n=85 | 0.502 |
| WtHR | 0.48 (0.45,0.51)  n=101 | 0.48 (0.45,0.51)  n=85 | 0.492 |
| WtHR z-score | 0.16 (-0.26,0.75)  n=101 | 0.22 (-0.56,0.79)  n=85 | 0.687 |
| SBP (mmHg) | 102.00 (97.00,108.50)  n=93 | 102.00 (95.00,109.00)  n=83 | 0.517 |
| DBP (mmHg) | 62.00 (57.00,68.00)  n=93 | 61.00 (55.50,68.50)  n=83 | 0.986 |
| Glucose (mg/dL) | 81.0 (77.00,87.00)  n=94 | 83.00 (75.75,87.00)  n=80 | 0.825 |
| Total Cholesterol (mg/dL) | 161.0 (150.0,182.0)  n=93 | 163.0 (144.2,183.5)  n=78 | 0.407 |
| HDL-c (mg/dL) | 54.0 (46.0,63.0)  n=93 | 55.0 (47.5,62.0)  n=79 | 0.273 |
| LDL-c (mg/dL) | 96.00 (85.00,102.60)  n=93 | 97.00 (77.00,112.45)  n=78 | 0.607 |
| Triglycerides (mg/dL) | 54.00 (46.00,70.00)  n=93 | 54.0 (44.0,65.0)  n=79 | 0.509 |
| Insulin (uU/mL) | 3.35 (2.00,5.16)  n=68 | 3.80 (2.00,5.80)  n=59 | 0.514 |
| HOMA-IR | 0.62 (0.44,1.02)  n=68 | 0.67 (0.41,1.22)  n=59 | 0.524 |
| CRP (mg/dL) | 0.06 (0.02,0.19)  n=84 | 0.08 (0.02,0.36)  n=69 | 0.746 |

Values are presented as median (interquartile range). *n* indicates the number of participants included in each analysis.

BMI: Body Mass Index; FMI: Fat Mass Index; FFMI: Fat-Free Mass Index; WC: Waist Circumference; WtHR: Waist-to-Height Ratio; SBP: Systolic Blood Pressure; DBP: Diastolic Blood Pressure; HDL-c: High-Density Lipoprotein cholesterol; LDL-c: Low-Density Lipoprotein cholesterol; HOMA-IR: Homeostatic Model Assessment of Insulin Resistance; CRP: C-Reactive Protein.

Table supplementary 2. Efficacy (per-protocol) analysis of Mediterranean lifestyle intervention on cardiovascular risk factors in girls.

|  | **Baseline** | | **12-month intervention** | | **∆ Intervention** | **∆ Control** | **Crude change** | **Effect size**  **(Intervention vs control)** | |
| --- | --- | --- | --- | --- | --- | --- | --- | --- | --- |
|  | **Intervention (n=56)** | **Control (n=47)** | **Intervention (n=47)** | **Control (n=37)** |  |  |  | **Estimate** | **p-value** |
| **BMI (kg/m^2^)** | 16.32 (15.44,17.27)  N=56 | 16.03 (15.09,16.98)  N=47 | 16.62 (15.66,18.01)  N=47 | 16.96 (15.94,18.41)  N=37 | 0.30 | 0.93 | 0.660 | -0.79 (-1.31-0.28) | **0.003** |
| **BMI z-score** | 0.50 (-0.14,1.06)  N=56 | 0.30 (-0.32,0.81)  N=47 | 0.68 (0.12,1.22)  N=47 | 0.72 (0.21,1.15)  N=37 | 0.18 | 0.42 | 0.211 | -0.43 (-0.71,-0.16) | **0.002** |
| **FMI (kg/m^2^)** | 3.85 (3.41,4.49)  N=40 | 3.52 (3.33,4.05)  N=34 | 4.03 (3.42,4.74)  N=34 | 4.06 (3.50,4.77) | 0.18 | 0.54 | 0.394 | -0.39 (-0.75,-0.03) | **0.033** |
| **FMI z-score** | 1.74 (1.16,2.54)  N=40 | 1.41 (1.04,1.98)  N=34 | 1.92 (1.19,2.78)  N=34 | 1.89 (1.24,2.85)  N=20 | 0.18 | 0.48 | 0.591 | -0.49 (-0.92,-0.05) | **0.028** |
| **FFMI** **(kg/m^2^)** | 12.06 (11.60,12.64)  N=40 | 11.63 (11.28,12.37)  N=34 | 11.92 (11.68,12.68)  N=34 | 12.26 (11.64,13.26)  N=20 | -0.14 | 0.63 | 0.350 | -0.25 (-0.86,0.36) | 0.491 |
| **FFMI z-score** | -1.31 (-1.88,-0.71)  N=51 | -1.63 (-2.04,-0.97)  N=42 | 0.45 (-0.21,1.36)  N=47 | 0.03 (-0.17,1.66)  N=36 | 1.76 | 1.66 | 0.101 | -0.52 (-1.22,0.17) | 0.139 |
| **WC (cm)** | 52.10 (49.68,54.10)  N=52 | 51.35 (48.62,54.42)  N=42 | 55.00 (52.50,58.50)  N=47 | 54.50 (52.50,58.00)  N=37 | 2.90 | 3.15 | 1.000 | -0.39 (-2.33,1.55) | 0.698 |
| **WtHR** | 0.49 (0.46,0.51)  N=52 | 0.47 (0.43,0.49)  N=42 | 1.61 (1.03,2.57)  N=47 | 1.35 (0.76,2.37)  N=35 | 1.12 | 0.88 | -0.179 | 0.16 (-0.65,0.97) | 0.694 |
| **WtHR z-score** | 0.43 (-0.24,0.86)  N=52 | 0.22 (-0.86,0.63)  N=42 | 1. (0.31,1.74)   N=47 | 0.72 (0.12,1.91)  N=37 | 0.57 | 0.5 | 0.444 | 1.01 (-0.47,0.68) | 0.729 |
| **SBP (mmHg)** | 101.00 (95.25,107.00)  N=47 | 99.00 (93.00,105.5)  N=41 | 102.25 (96.25,107.25)  N=47 | 104.5 (94.0,107.0)  N=37 | 1.25 | 5.5 | 1.25 | -0.16 (-4.48,4.15) | 0.938 |
| **DBP (mmHg)** | 63.0 (55.5,68.5)  N=47 | 60.0 (54.0,67.0)  N=41 | 63.0 (58.75,69.62)  N=47 | 63.0 (60.0,68.5)  N=37 | 0.0 | 3.0 | -3.000 | 1.17 (-3.08,5.43) | 0.584 |
| **Total Cholesterol (mg/dL)** | 160.5 (150.8,180.0)  N=48 | 163.5 (141.2,184.0)  N=38 | 160.0 (143.0,186.5)  N=43 | 165.5 (149.5,187.8)  N=32 | -0.5 | 2.0 | 6.000 | -2.33 (-15.78,11.12) | 0.729 |
| **HDL-C (mg/dL)** | 52.50 (48.25,59.25)  N=48 | 55.00 (48.00,60.75)  N=38 | 57.0 (47.5,62.0)  N=43 | 58.0 (47.75,65.50)  N=32 | 4.5 | 3.0 | 0.000 | 0.30 (-4.68,5.29) | 0.903 |
| **LDL-C (mg/dL)** | 95.40 (83.50,104.95)  N=48 | 96.00 (73.25,11.75)  N=38 | 91.0 (72.3,114.7)  N=43 | 95.5 (82.5,110.75)  N=32 | -4.4 | -0.5 | 2.800 | -3.21 (-15.28,8.85) | 0.595 |
| **Triglycerides (mg/dL)** | 56.50 (49.00,71.00)  N=48 | 59.00 (44.25,69.00)  N=38 | 63.0 (45.0,74.5)  N=43 | 67.00 (49.75,86.00)  N=32 | 6.5 | 8.0 | 5.000 | 2.30 (-13.25,17.86) | 0.767 |
| **Glucose (mg/dL)** | 81.0 (74.0,86.0)  N=49 | 79.5 (72.25,85.75)  N=38 | 84.0 (80.0,88.0)  N=43 | 83.0 (75.25,87.0)  N=32 | 3.0 | 3.5 | 0.500 | 1.57 (-2.62,5.77) | 0.454 |
| **Insulin (uU/mL)** | 3.45 (2.43,5.56)  N=34 | 4.20 (2.47,7.75)  N=28 | 3.03 (2.00,5.71)  N=32 | 4.30 (2.00,8.20)  N=23 | -0.42 | 0.1 | 0.300 | -4.00 (-10.84,2.83) | 0.241 |
| **HOMA-IR** | 0.63 (0.46,1.28)  N=34 | 0.83 (0.45,1.56)  N=28 | 0.62 (0.42,1.24)  N=32 | 0.88 (0.40,1.56)  N=23 | -0.01 | 0.05 | 0.066 | -1.02 (-2.76,0.70) | 0.235 |
| **CRP (mg/dL)** | 0.05 (0.03,0.25)  N=43 | 0.07 (0.03,0.29)  N=32 | 0.09 (0.03,0.50)  N=31 | 0.09 (0.04,0.26)  N=21 | 0.04 | 0.02 | 0.038 | 0.47 (-1.38,2.33) | 0.606 |

Values are presented as median (interquartile range). The level of statistical significance was fixed as p<0.05.

Analysis was adjusted by mother’s level education, adherence to the mediterranean diet in baseline, total energy intake, minutes of physical activity per week.

∆: change. BMI: body mass index; CRP: C-reactive protein; DBP: diastolic blood pressure; FMI: fat mass index; FFMI: fat free mass index; HDL-c: high-density lipoprotein cholesterol; HOMA-IR: homeostatic model assessment of insulin resistance; LDL-c: low-density lipoprotein cholesterol; SBP: systolic blood pressure; WC: waist circumference; WtHR: waist-to-height ratio.

Table Suplementary 3. Efficacy (per-protocol) analysis of Mediterranean lifestyle intervention on cardiovascular risk factors in boys.

|  | **Baseline** | | **12-month intervention** | | **∆ Intervention** | **∆ Control** | **Crude change** | **Effect size (Intervention vs control)** | |
| --- | --- | --- | --- | --- | --- | --- | --- | --- | --- |
|  | **Intervention (n=54)** | **Control (n=49)** | **Intervention (n=48)** | **Control (n=38)** |  |  |  | **Estimate** | **p-value** |
| **BMI (kg/m^2^)** | 15.95 (15.55,16.78)  N=54 | 15.98 (15.50,16.93)  N=49 | 16.11 (15.20,17.45)  N=48 | 16.30 (15.66,17.60)  N=38 | 0.16 | 0.32 | 0.030 | -0.14 (-1.08, 0.79) | 0.761 |
| **BMI z-score** | 0.28 (-0.18,0.86)  N=54 | 0.30 (-0.08,0.93)  N=49 | 0.44 (-0.31,1.08)  N=48 | 0.64 (0.12,1.35)  N=37 | 0.16 | 0.34 | 0.098 | -0.08 (-0.48, 0.32) | 0.697 |
| **FMI (kg/m^2^)** | 3.28 (2.92,3.90)  N=33 | 3.52 (3.19,3.71)  N=34 | 3.23 (2.86,3.80)  N=30 | 3.39 (3.13,4.62)  N=24 | -0.05 | -0.13 | -0.032 | -0.09 (-0.68,0.50) | 0.761 |
| **FMI z-score** | 1.50(1.20,2.73)  N=33 | 1.78 (1.45,2.49)  N=34 | 1.44 (0.83,2.13)  N=30 | 1.78 (1.43,3.62)  N=23 | -0.06 | 0.00 | 0.072 | -0.38 (-1.19,0.43) | 0.354 |
| **FFMI (kg/m^2^)** | 12.18 (11.61,13.19)  N=33 | 11.97 (11.49,12.60)  N=34 | 12.34 (11.77,12.98)  N=30 | 12.32 (11.70,13.07)  N=23 | 0.16 | 0.35 | 0.163 | -0.41 (-1.72,0.89) | 0.523 |
| **FFMI z-score** | -0.85 (-1.38,-0.25)  N=44 | -1.03 (-1.47,-0.49)  N=41 | -0.57 (-0.65,-0.02)  N=48 | -0.34 (-0.68,0.47)  N=38 | 0.28 | 0.69 | 0.054 | -0.13 (-0.51,0.23) | 0.464 |
| **WC (cm)** | 52.70 (51.00,56.20)  N=49 | 52.80 (50.80,55.25)  N=43 | 55.00 (53.00,58.30)  N=48 | 55.35 (52.50,59.76)  N=38 | 2.30 | 2.55 | -0.250 | 0.36 (-1.64,2.37) | 0.717 |
| **WtHR** | 0.47 (0.44,0.50)  N=49 | 0.48 (0.45,0.51)  N=43 | 0.93 (0.41,1.35)  N=48 | 1. (0.33,1.66)   N=38 | 0.46 | 0.52 | 0.030 | 0.21 (-0.47,0.90) | 0.532 |
| **WtHR z-score** | -0.91 (-1.82,-0.26)  N=49 | 0.22 (-0.41,0.92)  N=43 | 1.32 (0.51,1.47)  N=48 | 1.21 (0.31,1.91)  N=38 | 2.23 | 0.99 | -0.369 | 0.12 (-0.44,0.69) | 0.661 |
| **SBP (mmHg)** | 104.5 (97.25,109.38)  N=46 | 104.0 (97.0,110.0)  N=42 | 103.5 (94.0,111.8)  N=47 | 103.5 (98.5,111.0)  N=37 | -1.0 | -0.5 | -2.000 | -0.25 (-5.14,4.64) | 0.919 |
| **DBP (mmHg)** | 61.25 (57.62,68.0)  N=46 | 63.0 (57.5,72.75)  N=42 | 60.0 (54.5,63.0)  N=47 | 62.0 (57.0,67.0)  N=37 | -1.25 | -1.00 | -1.500 | -1.02 (-4.61,2.56) | 0.571 |
| **Total Cholesterol (mg/dL)** | 163.0 (149.0,183.0)  N=45 | 162.0 (144.8,181.2)  N=40 | 168.5 (144.8,181.0)  N=44 | 171.5 (152.8,191.2)  N=32 | 5.5 | 9.5 | -1.500 | -0.11 (-11.42,11.19) | 0.983 |
| **HDL-C (mg/dL)** | 55.0 (46.0,64.0)  N=45 | 56.0 (47.0, 62.0)  N=41 | 61.0 (50.0,71.0)  N=44 | 59.50 (53.75,69.25)  N=32 | 6.0 | 3.5 | -2.000 | 2.52 (-2.83,7.87) | 0.350 |
| **LDL-C (mg/dL)** | 96.00 (86.00,102.00)  N=45 | 97.40 (78.00,112.95)  N=40 | 96.00 (81.75,110.25)  N=44 | 99.0 (77.3,115.9)  N=31 | 0.0 | 1.6 | -1.700 | 1.24 (-8.33,10.82) | 0.796 |
| **Triglycerides (mg/dL)** | 52.0 (45.0, 64.0)  N=45 | 51.0 (43.0,62.0)  N=41 | 49.50 (38.00,61.75)  N=44 | 50.0 (43.5,68.0)  N=31 | -2.5 | -1.0 | 9.000 | -1.48 (-10.01,7.03) | 0.727 |
| **Glucose (mg/dL)** | 81.0 (78.0,89.0)  N=45 | 84.0 (79.0,89.5)  N=42 | 82.0 (77.75,88.25)  N=44 | 85.0 (81.5,90.0)  N=31 | 1.0 | 1.0 | 0.000 | -0.79 (-4.19,2.60) | 0.643 |
| **Insulin (uU/mL)** | 2.70 (2.0,4.17)  N=34 | 2.70 (2.0,5.4)  N=31 | 3.20 (2.0,4.83)  N=27 | 3.52 (2.0,6.0)  N=24 | 0.50 | 0.82 | 0.950 | -1.05 (-2.49,0.40) | 0.150 |
| **HOMA-IR** | 0.52 (0.44,0.90)  N=34 | 0.60 (0.40,1.12)  N=31 | 0.64 (0.41,1.00)  N=27 | 0.72 (0.42, 1.29)  N=24 | 0.12 | 0.12 | 0.179 | -0.25 (-0.59,0.09) | 0.146 |
| **CRP (mg/dL)** | 0.06 (0.02,0.18)  N=41 | 0.07 (0.02,0.67)  N=37 | 0.05 (0.02,0.50)  N=28 | 0.04 (0.02,0.06)  N=23 | -0.01 | -0.03 | 0.045 | 0.21 (-0.08,0.50) | 0.159 |

Values are presented as median (interquartile range). The level of statistical significance was fixed as p<0.05.

Analysis was adjusted by mother’s level education, adherence to the mediterranean diet in baseline, total energy intake, minutes of physical activity per week.

∆: change. BMI: body mass index; CRP: C-reactive protein; DBP: diastolic blood pressure; FMI: fat mass index; FFMI: fat free mass index; HDL-c: high-density lipoprotein cholesterol; HOMA-IR: homeostatic model assessment of insulin resistance; LDL-c: low-density lipoprotein cholesterol; SBP: systolic blood pressure; WC: waist circumference; WtHR: waist-to-height ratio.

Table Suplementary 4. Normalized Root Mean Square (NRMSE) values in the imputation process.

| **Variables** | **Girls** | **Boys** |
| --- | --- | --- |
| BMI (kg/m^2^) | 0.261 | 0.319 |
| BMI z-score | 0.455 | 0.493 |
| FMI (kg/m^2^) | 0.525 | 0.590 |
| FMI z-score | 0.503 | 0.608 |
| FFMI (kg/m^2^) | 0.294 | 0.365 |
| FFMI z-score | 0.425 | 0.443 |
| WC (cm) | 0.139 | 0.152 |
| WtHR | 0.487 | 0.500 |
| WtHR z-score | 0.457 | 0.519 |
| SBP (mmHg) | 0.130 | 0.145 |
| DBP (mmHg) | 0.225 | 0.220 |
| Glucose (mg/dL) | 0.149 | 0.128 |
| Total Cholesterol (mg/dL) | 0.227 | 0.208 |
| HDL-c (mg/dL) | 0.274 | 0.346 |
| LDL-c (mg/dL) | 0.358 | 0.270 |
| Triglycerides (mg/dL) | 0.502 | 0.451 |
| Insulin (uU/mL) | 0.998 | 0.720 |
| HOMA-IR | 0.480 | 0.452 |
| CRP (mg/dL) | 0.560 | 0.559 |

Normalized Root Mean Square Error (NRMSE) values represent the accuracy of the imputation process for each variable. Lower values indicate better imputation accuracy. BMI: body mass index; FMI: fat mass index; FFMI: fat-free mass index; WC: waist circumference; WtHR: waist-to-height ratio; SBP: systolic blood pressure; DBP: diastolic blood pressure; HDL-c: high-density lipoprotein cholesterol; LDL-c: low-density lipoprotein cholesterol; HOMA-IR: homeostatic model assessment of insulin resistance; CRP: C-reactive protein.Supplementary

Table Supplementary 5. Descriptive analysis of the sample that dropped out before the first year of assessment.

|  | Intervention (n=110) | | Control (n=96) | |
| --- | --- | --- | --- | --- |
|  | **Complete (n=95)** | **Drop out (n=15)** | **Complete (n=75)** | **Drop out (n=21)** |
| General characteristics | | | | |
| Sex (girls) | 47 (49.5%) | 6 (60.0%) | 37 (49.4%) | 10 (47.6%) |
| Age | 6.23±1.20 | 4.45±1.12 | 6.30±1.08 | 4.50±0.89 |
| Mediterranean diet adherence score | 13.01±2.31 | 9.71±2.21 | 10.84±2.82 | 11.25±3.51 |
| Mediterranean diet adherence** |  |  |  |  |
| Adherence (≥10 points) | 90 (94.8%) | 4 (26.7%) | 50 (66.7%) | 8 (38.1%) |
| Non-adherence (<10 points) | 5 (5.2%) | 11 (73.3%) | 25 (33.3%) | 13 (61.9%) |
| Total energy intake (kcal/day) | 1553.72±434.97 | 1882.09±487.15 | 1589±422.40 | 1893.35±703.56 |
| Physical activity (min/week) | 268.22±154.92 | 250.1±215.20 | 254.44±137.73 | 333.33±210.78 |
| Anthropometric and body composition | | | | |
| BMI (kg/m^2^) | 16.41 (15.43,17.55) | 16.10 (14.46,18.50) | 16.68 (15.79,17.70) | 15.92 (14.37, 17.81) |
| BMI z-score | 0.57 (-0.06,1.15) | 0.23 (-1.00, 1.92) | 0.64 (0.14,1.34) | 0.18 (-0.91, 1.52) |
| BMI categories |  |  |  |  |
| Under/normal weight | 69 (72.6%) | 11 (73.3%) | 54 (72.0%) | 19 (90.5%) |
| Overweight | 22 (23.2%) | 4 (26.7%) | 14 (18.7%) | 2 (9.5%) |
| Obesity | 4 (4.2%) | - | 7 (9.3%) | - |
| FMI (kg/m^2^) | 3.61 (3.07, 4.45) | 4.60 (2.42, 5.92) | 3.74 (3.34,4.77) | 3.19 (2.59, 3.66) |
| FMI z-score | 1.65 (1.06,2.52) | 3.10 (-0.09, 4.91) | 1.88 (1.39,3.06) | 1.27 (0.65, 1.82) |
| FFMI (kg/m^2^) | 12.08(11.70,12.88) | 12.67 (11.70, 13.54) | 12.32 (11.64,13.26) | 11.61 (11.19, 12.61) |
| FFMI z-score | -0.12 (-0.62,0.69) | -1.03 (-3.15, 0.64) | -0.08 (-0.54,0.72) | -1.61 (-2.67, -0.49) |
| WC (cm) | 55.00 (52.74,58.35) | 52.68 (46.0, 59.0) | 55.20 (52.50,58.35) | 50.90 (43.80, 56.30) |
| WtHR | 1.25 (0.62,2.09) | 0.49 (0.45, 0.52) | 1.11 (0.69,2.06) | 0.47 (0.41, 0.60) |
| WtHR z-score | 1.15 (0.40,1.58) | 0.56 (-0.26, 1.15) | 1.00 (0.21,1.92) | 0.03 (-2.15, 2.60) |
| Cardiovascular risk factors | | | | |
| SBP (mmHg) | 102.50 (95.75,109.00) | 111.37 (104.0, 119.0) | 104.25 (96.62,111.00) | 104.94 (85.0, 135.50) |
| DBP (mmHg) | 61.00 (57.00,68.00) | 75.12 (68.0, 87.0) | 62.75 (58.25,68.00) | 67.38 (52.50, 89.0) |
| Total Cholesterol (mg/dL) | 166.0 (144.5,184.0) | 169.14 (134.0, 209.0) | 167.0 (149.8,190.2) | 147.88 (120.0, 190.0) |
| HDL-C (mg/dL) | 58.0 (49.0,66.0) | 60.86 (45.0, 100.0) | 58.50 (50.00,67.25) | 48.33 (34.0, 62.0) |
| LDL-C (mg/dL) | 95.0 (78.0,111.7) | 102.71 (71.0, 134.0) | 97.0 (80.5,111.0) | 86.44 (70.80, 119.0) |
| Triglycerides (mg/dL) | 55.0 (41.5,68.5) | 62.85 (45.0, 95.0) | 56.0 (46.0,75.0) | 56.77 (31.0, 86.0) |
| Glucose (mg/dL) | 84.0 (79.0,88.0) | 82.0 (70.0, 91.0) | 84.0 (79.5,88.0) | 78.4 (64.0, 88.0) |
| Insulin (uU/mL) | 3.09 (2.00,5.37) | 6.90 (6.50, 7.30) | 3.76 (2.00,7.05) | 4.82 (2.0, 7.50) |
| HOMA-IR | 0.64 (0.42,1.21) | 1.48 (1.40, 1.57) | 0.72 (0.42,1.51) | 0.99 (0.36, 1.54) |
| CRP (mg/dL) | 0.08 (0.03,0.50) | 0.27 (0.00, 0.96) | 0.05 (0.03,0.25) | 0.78 (0.03, 2.80) |

Values are presented as n and % for categoric variables, mean ± SD and median (interquartile range) for quantitative variables depending on their distribution.

** Mediterranean diet adherence evaluated through validated MED4CHILD questionnaire ^25^

BMI categories according to Cole et al criteria ^19^

BMI: body mass index; CRP: C-reactive protein; DBP: diastolic blood pressure; FMI: fat mass index; FFMI: fat free mass index; HDL-c: high-density lipoprotein cholesterol; HOMA-IR: homeostatic model assessment of insulin resistance; LDL-c: low-density lipoprotein cholesterol; OB: obesity; OW: Overweight; SBP: systolic blood pressure; WC: waist circumference; WtHR: waist-to-height ratio.
